# Quality Analysis of Minerals Formed by Jaw Periosteal Cells under Different Culture Conditions

**DOI:** 10.3390/ijms20174193

**Published:** 2019-08-27

**Authors:** Marina Danalache, Sophie-Maria Kliesch, Marita Munz, Andreas Naros, Siegmar Reinert, Dorothea Alexander

**Affiliations:** 1 Department of Orthopedic Surgery, University Hospital, 72076 Tübingen, Germany; 2 Quality Analysis GmbH, 72622 Nürtingen, Germany; 3 Department of Oral and Maxillofacial Surgery, University Hospital, 72076 Tübingen, Germany

**Keywords:** bone mineral formation, fetal calf serum, human platelet lysate, osteoprogenitor jaw periosteal cells, mechanical properties, atomic force microscopy, Raman spectroscopy

## Abstract

Previously, we detected a higher degree of mineralization in fetal calf serum (FCS) compared to serum-free cultured jaw periosteum derived osteoprogenitor cells (JPCs). By Raman spectroscopy, we detected an earlier formation of mineralized extracellular matrix (ECM) of higher quality under serum-free media conditions. However, mineralization potential remained too low. In the present study, we aimed to investigate the biochemical composition and subsequent biomechanical properties of the JPC-formed ECM and minerals under human platelet lysate (hPL) and FCS supplementation. JPCs were isolated (*n* = 4 donors) and expanded under FCS conditions and used in passage five for osteogenic induction under both, FCS and hPL media supplementation. Raman spectroscopy and Alizarin Red/von Kossa staining were employed for biochemical composition analyses and for visualization and quantification of mineralization. Osteocalcin gene expression was analyzed by quantitative PCR. Biomechanical properties were assessed by using atomic force microscopy (AFM). Raman spectroscopic measurements showed significantly higher (*p* < 0.001) phosphate to protein ratios and in the tendency, lower carbonate to phosphate ratios in osteogenically induced JPCs under hPL in comparison to FCS culturing. Furthermore, higher crystal sizes were detected under hPL culturing of the cells. With respect to the ECM, significantly higher ratios of the precursor protein proline to hydroxyproline were detected in hPL-cultured JPC monolayers (*p* < 0.001). Additionally, significantly higher levels (*p* < 0.001) of collagen cross-linking were calculated, indicating a higher degree of collagen maturation in hPL-cultured JPCs. By atomic force microscopy, a significant increase in ECM stiffness (*p* < 0.001) of FCS cultured JPC monolayers was observed. The reverse effect was measured for the JPC formed precipitates/minerals. Under hPL supplementation, JPCs formed minerals of significantly higher stiffness (*p* < 0.001) when compared to the FCS setting. This study demonstrates that hPL culturing of JPCs leads to the formation of an anorganic material of superior quality in terms of biochemical composition and mechanical properties.

## 1. Introduction

Bone tissue engineering (BTE) has been continuously developing as a multidisciplinary field involving innovative biomaterials, mesenchymal stromal/stem cells and activating factors with the overall aim to restore and regenerate bone defects. Jaw periosteum derived progenitor cells (JPCs) are emerging as promising candidates in the field of maxillofacial reconstruction [1,2] due to their relatively easy availability, multipotency capacity at singular cellular level [3] and high proliferation rates [4].

To realize cell-based clinical applications using JPCs, a detailed characterization of the cells, their expression patterns and formed extracellular matrix, coupled with optimized culture and defined differentiation conditions are key factors to ensure the success of BTE constructs. Since fetal calf serum (FCS) supplementation of cell cultures is no longer up-to-date, especially when in vitro cultivated mesenchymal stem cells (MSC) are intended to be used for therapeutic purposes, human platelet lysate (hPL) has been taken into consideration as a suitable and promising alternative to FCS [5,6]. Additionally, the transmission of animal components and potential immune responses should be avoided. For hPL manufacturing, platelets have to be lysed for growth factor release from the contained alpha granules [6,7]. Moreover, several studies have shown that media supplemented with hPL actually shortened MSC expansion time while maintaining the cells phenotype and differentiation capacities [8,9,10]. Despite the aforementioned advantages, it must be taken into consideration that hPL has similar limitations as the use of FCS: There is no precise characterization of its entire composition, it is seldom distributed commercially, whereby the number of supplier companies is increasing and hPL might facilitate the transmission of human diseases [11]. In order to minimize the contamination risks, it has been recommended to store hPL for three months (quarantine time) and reanalyze afterwards for possible serum conversions [12]. The variations in composition can be addressed by using a large number of donors (20–120) [13].

In a scientific era in which static and two-dimensional culture conditions become obsolete and three-dimensional cell culture/bioreactors/3D-printing/microfluidic organ-on-a-chip systems are emerging, the demand of optical imaging approaches for live-monitoring of these systems, increases continuously [14]. Suitable optical technologies should be tested and improved in order to assure quality assessment of formed cell aggregates/tissue engineered constructs/small organs. In our opinion, Raman spectroscopy and atomic force microscopy (AFM) should be combined in order to monitor biochemical composition and stiffness and therefore the maturity of resulting bone-like extracellular matrix (ECM) formed by JPCs in vitro.

Raman spectroscopy has been emerging as a powerful tool in elucidating the biological compositions and signatures with a wide spectrum of applications, ranging from various pathological conditions [15,16,17,18] to tissues and cells [19,20]. Raman spectral studies have already been performed on structural analysis of various extracellular matrix (ECM) components [21,22,23,24].

Moreover, this technology has been successfully shown to provide a nondestructive readout of collagen-containing ECM in a medium-throughput culture system [25]. Additionally, we previously showed by Raman spectroscopy that the biochemical composition of crystals formed extracellularly differs under cultivation of JPCs without and with fetal calf serum (FCS).

The ECM not only provides structural support or anchoring of cellular components, but it also initiates and modulates the biomechanical signals required for the regulation of cellular behavior, such as differentiation, proliferation and migration [26,27,28]. The mechanical features of the JPC niche and implicitly its surrounding microenvironment are of particular relevance, especially in the field of bone constructs, as they should uphold and withstand continuous stretch/strain mechanisms. Atomic force microscopy (AFM) is a powerful tool that enables the investigation of local mechanical properties with a nanoscale resolution. This technology has been suggested to be a suitable and reliable technique with a large repertoire of applications [29]. Moreover, mechanical testing can be performed in situ under liquid physiological conditions, making it an appropriate approach to investigate local micromechanical properties, such as those of the ECM [30]. LeBlon et al. indicated that percentages of positively stained cells for osteogenic markers (osteopontin, osteocalcin) negatively correlated with increasing cell elastic modulus as detected by the Pearson correlation coefficient [31]. Therefore, an increasing elastic modulus led to decreased osteogenic differentiation. In accordance to these findings, it has been demonstrated that human MSCs undergoing osteogenesis develop a decreased cytoskeleton elasticity, which was attributed to the reorganization/arrangement of the actin network during differentiation [32].

In a previous study, we performed phenotypic and functional characterization of JPCs under FCS and hPL supplementation and detected significantly higher proliferation and metabolic activities as well as higher mineralization potential of JPCs under hPL conditions [33]. In the present study, we extend this knowledge and examine biochemical composition and mechanical properties of the formed matrix during in vitro osteogenesis under FCS and hPL medium supplementation using the high precision techniques of Raman spectroscopy and atomic force microscopy.

## 2. Results and Discussion

### 2.1. Analysis of JPC Mineralization under hPL and FCS Supplementation

**Figure 1:**
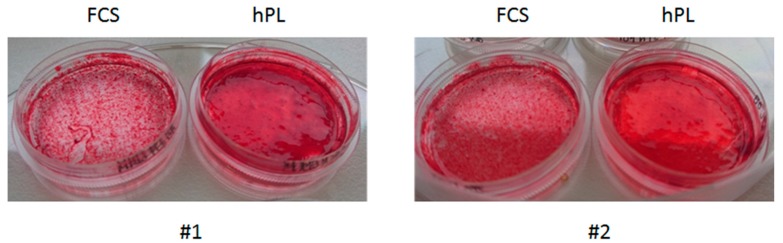
Different consistency and macroscopic appearance of osteogenically induced JPC monolayers following FCS and hPL medium supplementation (Alizarin Red staining). Representative images of cell monolayers from two donors (#1, #2) cultured in AFM petri dishes (plastic bottom) under both supplementations. Abbreviations: FCS—fetal calf serum, hPL—human platelet lysate.

In terms of JPCs mineralization degree/ECM appearance, representative microscopic images in Figure 1 show a different consistency of hPL-supplemented (right) and osteogenically induced JPC monolayers compared to the FCS-supplemented (left) counterparts derived from two donors (#1, #2). JPCs formed an ECM of gel-like viscosity and consistency and appeared to be thicker under hPL compared to FCS supplementation.

**Figure 2:**
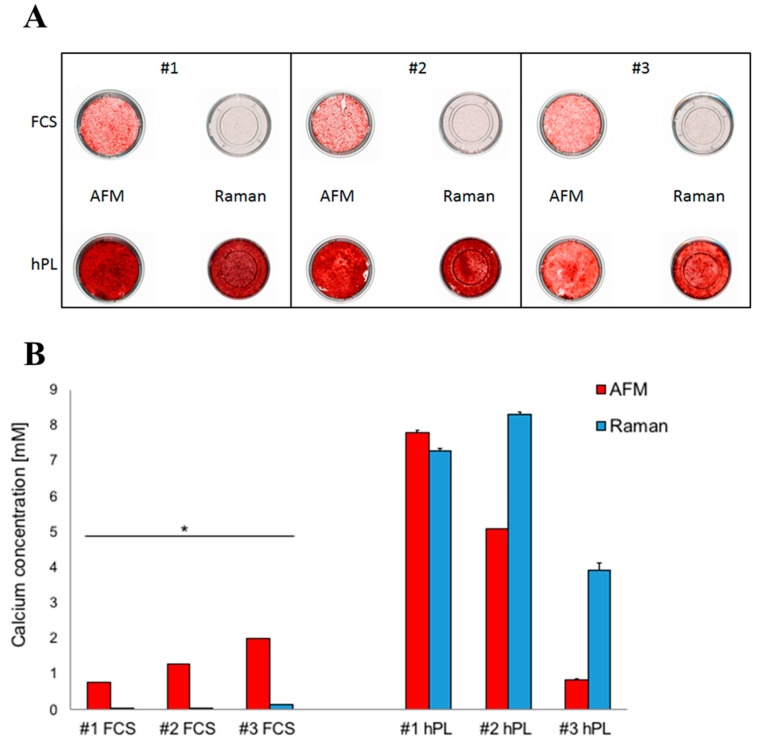
Detection of JPCs mineralization (Alizarin Red staining) in AFM and Raman petri dishes under FCS and hPL supplementation. Macroscopic images: JPC mineralization from three donors (#1, #2, #3) cultured in AFM (plastic bottom) and Raman (glass bottom) petri dishes was observed after osteogenic induction for 25 days under both medium supplementations (**A**). Quantification of JPC mineralization degrees in cell monolayers from the three donors (**B**). Calcium levels detected in cell monolayers cultured in AFM dishes are represented by red columns and those in Raman petri dishes by blue columns. Abbreviations: AFM—atomic force microscopy, FCS—fetal calf serum, hPL—human platelet lysate, * *p* < 0.05.

When JPCs were cultured in AFM and Raman glass dishes, respectively, FCS-cultured JPCs mineralized much better in AFM dishes compared to Raman ones, where cell mineralization occurred to a later time point (Figure 2A). In contrast, hPL-supplemented JPCs strongly mineralized in both culture plates. Interestingly, in two of the three tested patient cells (Figure 2—#2 and #3), Raman dishes seemed to further support cell mineralization of hPL-supplemented JPCs, as also confirmed by the subsequent quantification of the Alizarin staining (Figure 2B). The differences in calcium concentration detected in Raman and AFM dishes were highly significant for both FCS- and hPL-cultured JPCs. Under hPL supplementation, cell mineralization was shown to be significantly higher in cells derived from Figure 2 #1 and #2. Cells derived from patient Figure 2 #3 showed a significantly higher osteogenic potential under FCS supplementation particularly in AFM dishes (as shown in Figure 2B) in contrast to Raman dishes. Under hPL supplementation exactly the opposite was the case and JPCs in Raman dishes showed a significantly higher mineralization potential.

**Figure 3:**
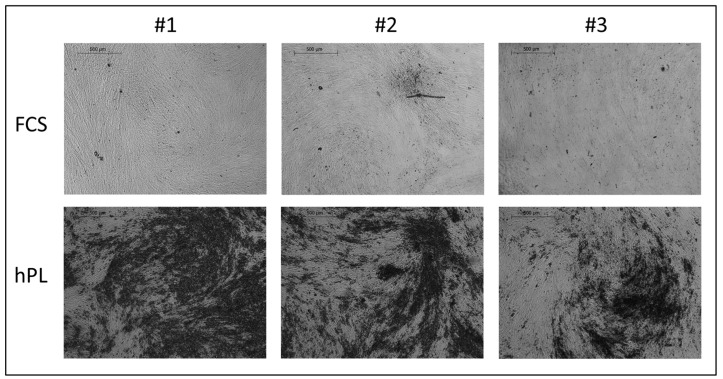
Detection of JPCs mineralization by von Kossa staining in Raman dishes under FCS and hPL supplementation. Microscopic images showing JPC mineralization from three donors (#1, #2, #3) cultured in Raman (glass bottom) dishes were observed after osteogenic induction for 25 days under both medium supplementations. Abbreviations: FCS—fetal calf serum, hPL—human platelet lysate.

JPCs cultured in Raman glass dishes were additionally stained by von Kossa after 35 days of osteogenic induction, as shown in Figure 3.

### 2.2. Detection of Osteocalcin Gene Expression Levels in JPCs Cultured under FCS and hPL Supplementation

**Figure 4:**
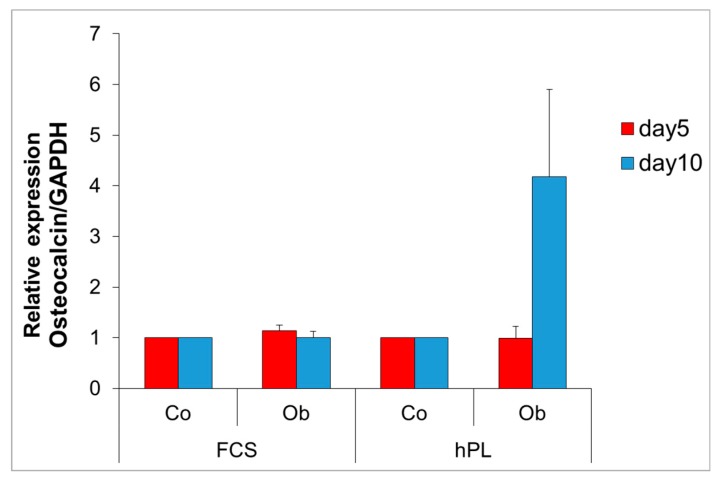
Relative gene expression levels of human osteocalcin transcript levels by quantitative PCR. JPCs from four donors were cultured in 75 cm^2^ culture flasks under normal (Co) and osteogenic (Ob) conditions for five and 10 days under both medium supplementations, following RNA isolation for osteocalcin gene quantification. Gene expression in untreated JPCs were set as 1 and induction indices in relation to untreated controls were calculated. Abbreviations: FCS—fetal calf serum, hPL—human platelet lysate.

JPCs from four donors were cultured under normal (Co) and osteogenic (Ob) culture conditions for five and 10 days under FCS and hPL supplementation. Osteocalcin transcript levels were shown to be highly upregulated (4.2-fold) in osteogenically-induced JPCs only in hPL-supplemented JPCs at day 10. However, due to high variations between the donor cells, differences did not reach significance (Figure 4).

### 2.3. Characterization of hPL- and FCS-Cultured JPC-Formed Extracellular Matrix ECM by Raman Spectroscopy 

**Figure 5:**
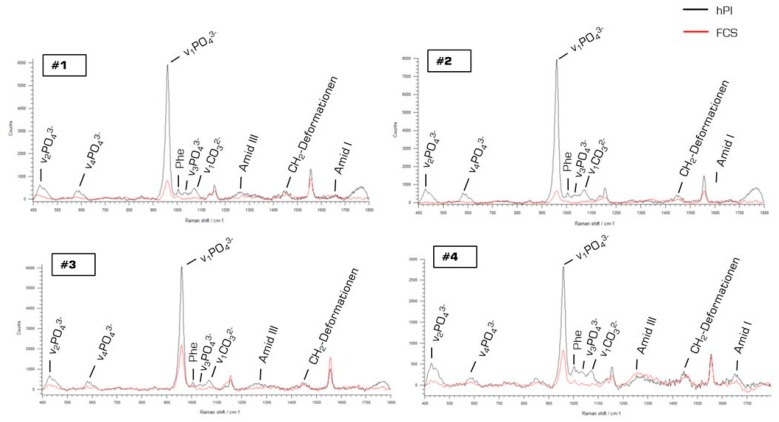
Mean Raman spectra from hPL- and FCS-supplemented JPCs. Mean Raman spectra from four donors (#1, #2, #3, #4) generated from JPCs growing in Raman glass dishes under hPL (black spectra) and FCS (red spectra) supplementation. Abbreviations: FCS—fetal calf serum, hPL—human platelet lysate.

Mean Raman spectra containing information about the biochemical composition of JPC-formed ECM (derived from four (#1–#4) donors) under osteogenic conditions and different supplementation (FCS versus hPL) are illustrated in Figure 5.

**Figure 6:**
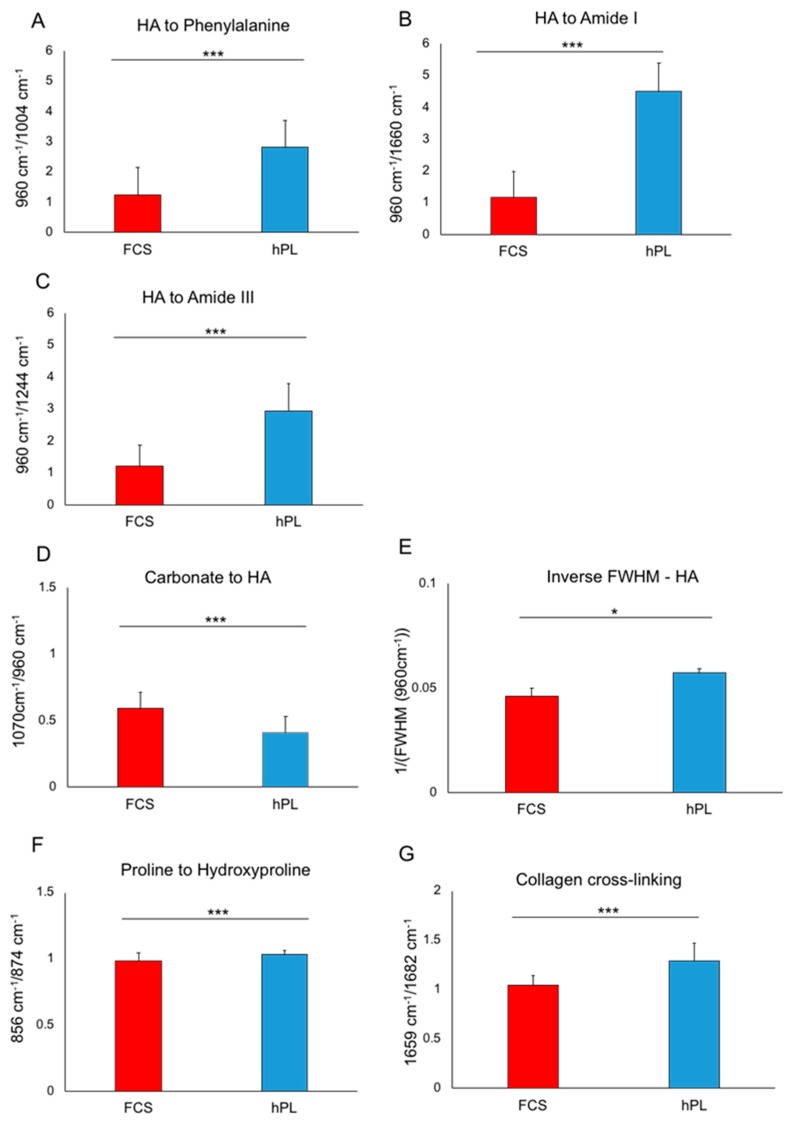
Ratios calculated from mean Raman spectra reflecting the biochemical composition of osteogenically-induced (OB) JPCs (four donors) under hPL and FCS supplementation. Phosphate to protein ratios (**A**) HA to phenylalanine; (**B**) HA to amid I; (**C**) HA to amid III; (**D**) carbonate content; (**E**) crystal size; (**F**) proline to hydroxyproline; (**G**) collagen cross-linking. Means (126–162 measurements per donor) ± standard deviations are depicted. * *p* < 0.05; *** *p* < 0.001. Abbreviations: FCS—fetal calf serum, hPL—human platelet lysate.

As illustrated in Figure 5, apart from the main hydroxyapatite peak at 960 cm^−1^, the three other phosphate peaks were shown to be higher under hPL, compared to FCS, supplementation. Further differences were detected in other peaks such as phenylalanine at 1004 cm^−1^, carbonate at 1070 cm^−1^, amid III at 1244 cm^−1^ and amid I at 1660 cm^−1^. From these Raman spectra and considering the mentioned peaks, different phosphate to protein ratios (HA to phenylalanine, HA to amid III, HA to amid I) were calculated by considering the respective wave numbers (as shown in Figure 6). Furthermore, the carbonate content (carbonate to phosphate) and the crystal size (reflected by the HA crystallinity: reverse of the full width of half maximum of the HA peak) were assessed in order to be able to evaluate the replacement of phosphate by carbonate and mineralization degree by comparing crystal sizes. Collagen matrix maturity was assessed by the calculation of proline to hydroxyproline with proline being the precursor of hydroxyproline which in turn is responsible for collagen fibril stability. Illustrated columns in Figure 6 depict means of 126–162 measurements per donor cells with four donors in total. We detected significantly higher HA to phenylalanine, HA to amid I and HA to III ratios (Figure 6A–C) in hPL-supplemented JPC monolayers indicating higher HA deposition in comparison to FCS-cultured JPCs. Furthermore, significantly lower carbonate content (Figure 6D) was calculated for the ECM from this group. JPCs cultured under hPL supplementation formed significantly larger crystals, as reflected in the diagram in Figure 6E by the means of FWHM values. Significant differences between the FCS- and hPL-group with calculated *p*-values are listed in Table 1.

### 2.4. Characterization of Mechanical Properties of hPL- and FCS-Cultured JPC-Formed ECM by Atomic Force Microscopy (AFM)

**Figure 7:**
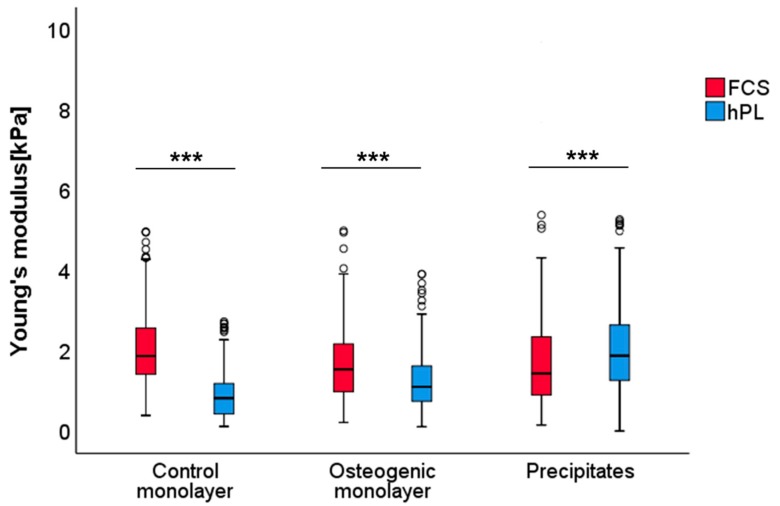
Analysis of Young’s modulus of JPC monolayers cultured under FCS and hPL supplementation. Boxplots (medians, minimum, maximum) of the stiffness (kPa) measured by atomic force microscopy for each analyzed group are depicted. In both, control (untreated) and osteogenic monolayers of FCS cultured monolayers analyzed ECM revealed higher stiffness when compared to hPL groups. The reversed effect was observed for the group of precipitates. *** *p* < 0.001 (for exact *p*-values, see Table 3). Abbreviations: FCS—fetal calf serum, hPL—human platelet lysate.

Osteogenically induced JPCs under FCS and hPL medium supplementation from four donors were subjected to AFM elasticity measurements of the ECM. Therefore, a total of 1122 measurements were performed. After exclusion of extreme values, a total of 330 measurements for the control group (FCS—169 and hPL—161), 339 for the osteogenic induced group (FCS—171 and hPL—168) and 417 measurements for the group of precipitates (FCS—211 and hPL—206) were included in the final analysis. The measured JPCs elastic moduli results are shown in Figure 7 and Table 3.

Stiffness of the ECM significantly increased under FCS culturing conditions when compared to hPL culturing settings for both the untreated controls (*p* < 0.001) and the osteogenic induced (*p* < 0.001) monolayers. In contrast, for the JPC-formed precipitates under hPL supplementation, a significant increase in stiffness (*p* < 0.001) was detected compared to the FCS group. Absolute values were thereby reduced by 57% under hPL culture conditions (median of 1.8 kPa for the FCS to 0.8 kPa for the hPL group) in control monolayers and by 28% (median of 1.5 kPa for the FCS to 1.1kPa for the hPL group) in osteogenic monolayers. As mentioned before, the reverse observation was made for the group of precipitates where a 24% increase of Young’s modulus was detected under hPL culture conditions (median of 1.4 kPa for the FCS to 1.9 kPa for the hPL group). Descriptive statistics and *p*-values for significant differences between the FCS- and hPL-group are listed in Table 2 and Table 3.

## 3. Discussion

Since the pioneering work of Nakahara and co-authors in the early 1990s, who explored the suitability of JPCs for bone tissue constructs applications [34], several studies have shown that they are comparable to, if not superior to bone marrow MSCs with respect to their bone healing and regeneration capacity [35,36]. For tissue engineering applications of jaw periosteal cells [1], a well-defined and clinical oriented approach has to be defined in terms of cell harvesting, serum-free culturing conditions and subsequent characterization has to be established. In the present study, we cultivated JPCs in hPL- and FCS-supplemented media in order to investigate the biochemical and biomechanical effects of different types of media on the JPC-formed ECM.

JPCs expanded under hPL supplementation grew significantly faster than the FCS-supplemented ones, as we published previously [33]. This might be indicative of upregulated S, G_2_/M phases as previously shown by Xia Et al. [37]. Additionally, we demonstrated in the same publication significantly higher mineralization degrees of JPCs expanded under hPL compared to FCS supplementation [33]. Even though the exact mechanism that triggers JPCs fast expansion and differentiation by hPL supplementation is not fully understood, it might be attributed to elevated concentrations of growth and differentiation factors contained in hPL. While the content of growth factors varies with hPL preparation, several studies emphasized EGF, bFGF, HGF, PDGF-AB, TGF-β1 and VEGF as being secreted by platelets [37,38,39]. Additionally, while a combination of the factors PDGF, bFGF and TGF-β1 was described as being “sufficient” to expand MSCs in a serum-free culture setting [40], the combination of PDGF, bFGF and IGF-1 has been shown to induce osteogenic differentiation of MSCs [41,42,43]. In order to improve in vitro JPC mineralization by optimization of cell culture conditions and for the simultaneous establishment of clinical-grade protocols, we tested in a further former study a serum-free medium for JPC cultivation and osteogenic differentiation. Analyses of the resulting phenotype and functional differences in comparison to FCS-supplemented cultures revealed higher proliferation activities and a selection of osteoprogenitor cells (MSCA-1 positive cells) under serum-free cultivation [44]. Raman analysis of the extracellular matrix showed a more mature mineralization and a higher quality of minerals formed under serum-free culture conditions [45]. Despite these advantages, mineralization degree remained too low, probably based partly on insufficient matrix component secretion such as type I collagen. In order to move a step further, we tested clinical-grade platelet lysate for JPC supplementation and detected, as already mentioned, significantly higher proliferation rates and mineralization potential of JPCs even without the addition of dexamethasone [33]. In the present study, we analyzed the biochemical composition and mechanical properties of the extracellular matrix synthesized under hPL supplementation in order to get a deeper understanding of JPC osteogenesis and in order to move forward to clinical applications. By Raman analyses, we detected higher phosphate to protein ratios indicating higher phosphate deposition under hPL supplementation. Under these conditions, carbonate content was found to be lower and crystal size to be significantly higher, thus reflecting a higher quality of formed crystals containing lesser carbonate and a stronger mineralization potential. Concerning the maturation process of collagen, a higher release of the precursor amino acid proline was detected while amounts of the mature hydroxyproline were proportionally lower under hPL, compared to FCS supplementation. Since hydroxyproline is responsible for collagen fibril stability, these findings could indicate a softer consistency of the collagen matrix, as already observed and illustrated in Figure 2 (gel-like appearance). However, collagen cross-linking seems to be significantly higher under hPL conditions, thus potentially conferring a higher tensile strength of the collagen matrix. Moreover, similar observations have been made by Gupta and co-authors. They showed in an in vivo setting after implantation of MSC-seeded calcium phosphate carriers in nude mice, a more mature mineralized tissue when MSCs were pre-expanded under hPL supplementation. FCS- expanded cells exhibited after implantation in nude mice the formation of a rather fibrous tissue [46].

It is known that MSCs differentiation characteristics and phenotypic manifestation are strongly regulated by ECM stiffness and density [47]. The arising question in our study was whether biochemical signals induced by hPL and FCS media supplementation could be transferred into physical mechanical properties reflecting mineralization capability of the JPCs. As indicated by our AFM data, the JPC-ECM exhibited a significant stiffness increase (*p* < 0.001) for control and osteogenically-induced monolayers under FCS when compared to hPL supplementation. In contrast, in terms of JPC-formed precipitates under hPL conditions, a significant stiffness increase (*p* < 0.001) was detected compared to FCS culturing settings. As shown by Roberts and co-authors, even though the FCS supplementation of periosteal cells has a positive effect on the osteocalcin and alkaline phosphatase (APL) expression in the early stages of osteogenic differentiation, further osteoinductive agents are required for the terminal differentiation into mature osteoblasts, such as: trans-retinoic acid, dexamethasone and bone morphogenic protein 2 (BMP2) [48]. Platelet lysate contains a potpourri of factors platelets are composed of, and therefore superior to xenogenic serum [49].

A relatively large variation in the Young’s moduli of JPC-formed EMC was detected, even within the same subgroups. This may reflect partially the heterogeneity of the cells. Furthermore, since a polymeric bead of ~25 µm was used for AFM indentation of ECM regions present in close vicinity of the cells, local structural variations such as the presence of stress fibers and distribution of various cytoskeleton elements (i.e., actin filaments, microtubules and intermediate filaments) might contribute to the increase in overall stiffness variability. This phenomenon was already described two decades ago [50].

## 4. Material and Methods

### 4.1. Cell Isolation and Culture of Jaw Periosteal Cells (JPCs)

JPCs derived from 4 donors were included in this study in accordance with the local ethical committee (approval number 618/2017BO2; date (15.12.2017 and 26.03.2019) and after obtaining written informed consent for the participants. The jaw periosteal tissue was cut in small pieces with a scalpel and enzymatically digested with type XI collagenase (1500 U/ml, Sigma-Aldrich, Steinheim, Germany) for 90 min. Enzymatically isolated cells were expanded in DMEM/F12 + 10% fetal calf serum (FCS) for up to 4 passages until used in passage 5–6 for osteogenic differentiation experiments. Detailed phenotypic and functional characterization of arisen JPCs under FCS and hPL supplementation was published previously by our group [33]. These cells represent the population of JPCs targeted and analyzed in the present study. For Raman measurements, glass bottom dishes (Cellview cell culture dishes from Greiner Bio-One GmbH, Germany) were used for JPC culturing and measuring. For AFM analyses, JPCs were cultured and measured in tissue culture dishes (TPP, Trasadingen, Switzerland). In the following, the terms Raman and AFM dishes will be used in order to distinguish between both culture plates. Comparison of the biochemical composition and mechanical properties of cell-formed extracellular matrix followed under DMEM/F-12 (Invitrogen-BioSource Europe, Nivelles, Belgium) culturing containing 10% FCS (Sigma-Aldrich, Steinheim, Germany) or 10% platelet lysate, both containing 1% amphotericin B and penicillin/streptomycin (Biochrom, Berlin, Germany). The used hPL was provided by the Centre for Clinical Transfusion Medicine in Tübingen, it did not contain heparin and was referred to as a research lysate based on the absent quarantine period. DMEM-cultured cells were passaged using trypsin-versene EDTA (1x, Lonza, Basel, Switzerland) and medium change was performed three times per week.

Osteogenic conditions were performed for all experiments by the addition of dexamethasone (4 µM), β-glycerophosphate (10 mM) and L-ascorbic acid 2-phosphate (100 µM) for at least 20 days. Due to the fact that especially FCS-supplemented JPCs mineralized in AFM dishes much more quickly than in Raman dishes, we chose different time points for the respective Raman and AFM measurements. 

### 4.2. Quantification of Cell Mineralization by Alizarin Dye Staining

hPL- and FCS-supplemented JPCs from 3 donors were induced osteogenically in Raman and AFM dishes (*n* = 3 per culture condition, untreated and osteogenic) for at least 20 days and cell monolayers were fixed with 4% formalin for 20 min. After two wash steps with PBS, 1 mL of a 40 mM Alizarin dye solution with a pH of 4.2 was added to the monolayers for 20 min while shaking. Unbound dye was washed 4 times with distillated water for 15 min. Alizarin dye was dissolved out from the monolayers by the addition of 10% acetic acid solution, for 20 min while shaking. Cell layers were detached by scraping and pipetted into 1.5 mL tubes for heating at 85 °C for 10 min while vigorously mixing. After cooling the samples on ice for 5 min and centrifugation at 20,000 *g* for 20 min, supernatants were neutralized by the addition of 10% ammonium hydroxide. The standard curve for the calculation of calcium concentrations in the samples was plotted as a function of a serially diluted (1:2 dilution) Alizarin Red stock solution (40 mM) to standard concentrations between 2 mM and 0.031 mM. Photometrical measurements were performed at a wavelength of 405 nm using the ELx800 photometer (BioTek Instruments GmbH, Bad Friedrichshall, Germany). Additionally, JPCs grown within Raman dishes were also stained by von Kossa. Therefore, cell monolayers were cold-fixed with 100% ethanol for 15 min and washed with distilled water three times before incubation with 5% silver nitrate for 1 hour at room temperature. After washing, sodium carbonate (0.05 g/mL) in a 9% formalin containing solution was added for 2 min and finally sodium thiosulfate (0.05 g/mL) was added for an additional 2 min of incubation. After three wash steps, images were taken in the water solution. 

### 4.3. Raman Spectroscopy Measurements of hPL- and FCS-Supplemented JPC Monolayers

An inVia Qontor Raman microscope (Renishaw GmbH, Pliezhausen, Germany) was employed for all measurements by Sophie-Maria Kliesch at the company Quality Analysis (Nürtingen, Germany). Raman spectra were excited by a 785 nm laser beam through a 40× water-immersion objective (Leica Microsystems GmbH, Wetzlar, Germany). The system was calibrated based on the silicon peak at 520 cm^−1^ prior to all measurements. The laser output power was set on 150 mW (due to scattering losses, only 50% of the laser power reaches the sample) for spectra acquisition. Raman spectra were collected from either JPCs cultured with serum or human platelet lysate containing media (DMEM/F12), after visible and robust cell mineralization for 20–30 days of osteogenic induction. JPCs from 4 donors were cultured for Raman measurements in uncoated glass bottom cell culture dishes (Greiner Bio-One GmbH, Frickenhausen, Germany), in the following referred to as Raman dishes. The total acquisition time per spectrum was 3 seconds within a mapping measurement with 126–162 single spectra. A background spectrum from the glass dish containing the cell culture medium was taken for each set of data. All acquired Raman spectra were background-subtracted using the specific background spectrum and then baseline corrected (arithmetic operation) using WiRE (Renishaw GmbH, Pliezhausen, Germany). A smoothing algorithm (Savitzky–Golay, second polynomial order, 7 data points) was employed on all spectra.

Raman spectra were investigated to compare the biochemical composition of JPC-formed extracellular matrix under FCS and hPL supplementation. For this purpose, the following spectral ratios were calculated from the Raman spectra: hydroxyapatite (HA) to phenylalanine (960/1004 cm^−1^), HA to amide I (960/1660 cm^−1^), HA to amide III (960/1244 cm^−1^) and carbonate to HA (1070/960 cm^−1^). For the calculation of HA crystallinity (crystal size), the inverse of the full-width half maximum (FWHM) was taken into consideration for the spectral range from 900–1000 cm^−1^ using WiRE software. The apatite peak was fitted using a standard Gaussian curve. Based on the fitted curve the inverse of FWHM (1/FWHM) was calculated for the HA peak at 960 cm^−1^.

### 4.4. Biomechanical Characterization of hPL- and FCS-Supplemented JPC Monolayers by Atomic Force Microscopy (AFM)

**Figure 8:**
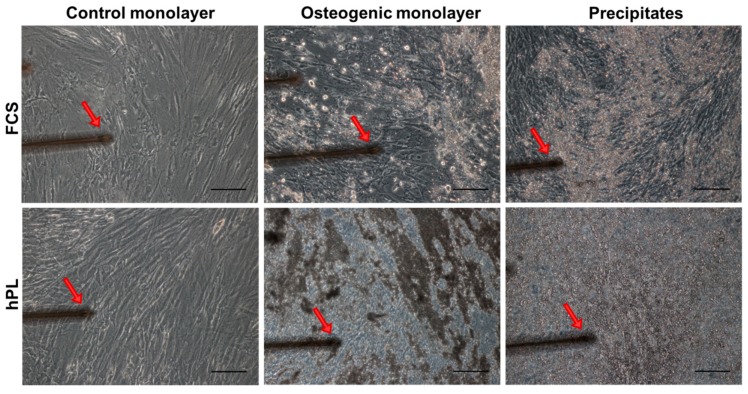
Representative microscopic images indicating the regions of interest subjected to elasticity measurements. Microscopic pictures of AFM measured JPCs—control monolayers (left pictures), osteogenic induced JPCs (middle) and calcium phosphate precipitates (right pictures) formed under hPL and FCS culturing conditions, respectively. Red arrows depict the position of the cantilever for elasticity measurements of non-mineralized regions in untreated (left) and osteogenic monolayers (middle, without precipitates) and of calcium phosphate precipitates. Images were acquired with the inverted AxioObserver D1 light microscope attached to the AFM system at a 10× magnification. Scale bar (black) represents 30 µm. Abbreviations: AFM—atomic force microscopy, JPCs—jaw periosteum derived progenitor cells, FCS—fetal calf serum, hPL—human platelet lysate.

Elastic moduli of JPCs were assessed (*n* = 4 donors) using an AFM system (CellHesion 200, JPK Instruments, Berlin, Germany) mounted on to an inverted light microscope (AxioObserver D1, Carl Zeiss Microscopy, Jena, Germany), which allowed simultaneous visualization of the cells. This modular approach allowed us to position and measure specific regions of interest within the dishes. Calibration of the cantilever was done on a clean surface of a petri dish; it was performed on the retracted curve and the spring constant was determined by using the thermal noise method incorporated into the device software (JPK Instruments, Berlin, Germany). Measurements were performed in force spectroscopy mode by recording single force–distance curves at the position of interest without laterally scanning the sample. For microscale indentation, a polymeric microsphere (25 µm in diameter, Polysciences, Inc., Warrington, PA, USA) was glued (M-Bond 610 Adhesive, Micro-Measurements, Raleigh, NC, USA) to an AFM cantilever (tip A, *k* = 0.2 N/m, All-In-One-Al-Tl, Budget Sensors, Sofia, Bulgaria). Indentation curves were sampled at 2 kHz, with a force trigger of ~10 nN and a velocity of 5 µm/sec. To evaluate elastic properties of the JPC-formed ECM under FCS and hPL culturing conditions, we applied indentations over the chosen region of interest identified by microscopic examination (~50 different positions/culture condition) (Figure 8). The Young’s modulus is the ratio of uniaxial force per unit surface in pascal divided by the adimensional proportional deformation of the examined sample and was calculated from the force–distance curves by using the Hertz fit model incorporated in the data processing software (JPK Instruments, Berlin, Germany).

### 4.5. Osteocalcin Gene Expression by Quantitative PCR

Untreated and osteogenically induced JPCs under FCS and hPL supplementation from 4 donors were cultured for 5 and 10 days before RNA isolation followed using the RNeasy Mini kit following manufacturer’s instructions. RNA concentration was determined using fluorescent staining (RNA HS Assay kit from ThermoFisher Scientific, Waltham, MA, USA) and measurement with a Qubit 3.0 fluorometer (Life Technologies/ThermoFisher Scientific, Waltham, MA, USA). 100 ng RNA was used for cDNA synthesis with the SuperScript^TM^ Vilo^TM^ Master Mix (Invitrogen/ThermoFisher Scientific, Waltham, MA, USA). For PCR reactions, commercially available primer kits from Search-LC (Heidelberg, Germany) and the DNA Master SybrGreen I kit (Roche, Basel, Switzerland) were used. PCR amplifications were performed with the LightCycler System (Roche, Mannheim, Germany) following a touchdown PCR protocol (annealing temperature between 68 and 58 °C). Osteocalcin transcript levels were normalized to those of the house keeping gene glycerinaldehyde 3-phosphate dehydrogenase (GAPDH). Ratios of osteocalcin/GAPDH in untreated JPCs were set as 1 and induction indices of osteogenically induced samples were calculated.

### 4.6. Statistical Analysis

Normality of the data was assessed by means of Shapiro–Wilks tests and histograms. Based on normality, for both the Raman and AFM data sets, the non-parametric Mann–Whitney U test was used to determine whether the differences between the groups were significant or not. Raman values are displayed as means ± standard deviations (*p*-values are listed in Table 1), while AFM values are presented as median (minimum–maximum, *p*-values in Table 2) and graphically displayed as boxplots. Additionally, for the AFM results, means, standard deviations, and standard errors of the mean are additionally listed in Table 3. In terms of calcium quantification (Alizarin Red staining) data are shown as means ± standard deviations and a two-tailed Student’s *t*-test was used to determine statistically significant differences between the groups. Statistical analysis was performed using the SPSS Statistics 22 (IBM Corp., Armonk, NY, USA) software. A *p*-value < 0.05 was considered significant.

## 5. Conclusions

In summary, nanoindentation combined with the Raman technology represent an exciting tool for the biochemical and mechanical examination of bone-like matrix formed by osteogenically-induced JPCs. By using these technologies, we detected higher degrees of mineralization in hPL compared to FCS supplemented monolayers, based on higher phosphate to matrix ratios and increased size and stiffness of calcium phosphate precipitates. Therefore, hPL supplementation not only accelerates JPCs proliferation activities as already demonstrated in our previous study [33], but also leads to the formation of anorganic material of superior quality in terms of biochemical composition and mechanical properties.

## Figures and Tables

**Table 1:** Calculation of significant differences of Raman ratios in FCS/hPL groups.

Ratios	*p*-Values
	FCS/hPL
HA to Phenylalamine	<0.001
HA to Amide I	<0.001
HA to Amide III	<0.001
Carbonate to HA	<0.001
Inverse FWHN–HA	0.029
Proline to hydroxyproline	<0.001
Collagen cross-linking	<0.001

Mann–Whitney U test with calculated *p*-values. Abbreviations: FCS—fetal calf serum, hPL—human platelet lysate, HA—hydroxyapatite.

**Table 2:** Calculation of significant differences of Young’s moduli detected in untreated (control) and osteogenic monolayers and of calcium phosphate precipitates in FCS/hPL groups.

Groups	*p*-Values
	FCS/hPL
Control monolayer	<0.001
Osteogenic monolayer	<0.001
Precipitates	<0.001

Mann–Whitney U test with calculated *p*-values. Abbreviations: FCS—fetal calf serum, hPL—human platelet lysate.

**Table 3:** Descriptive statistics of Young’s moduli in untreated (control) and osteogenic monolayers and of calcium phosphate precipitates in FCS/hPL groups.Medians with minimum and maximum, means, standard deviations and standard errors of mean are depicted.

Descriptive Statistics	Control Monolayer		Control Monolayer		Precipitates	
	FCS	hPL	FCS	hPL	FCS	hPL
Median	1.868	0.820	1.538	1.102	1.433	1.875
Minimum	0.390	0.116	0.214	0.109	0.151	0.003
Maximum	4.966	2.728	4.992	3.913	362.826	88.979
Mean	2.081	0.961	1.663	1.297	10.385	5.114
Standard deviation	0.994	0.671	0.907	0.774	44.799	12.688
Standard error	0.076	0.052	0.693	0.597	0.884	3.084
